# Performance of Machine Learning-Based Multi-Model Voting Ensemble Methods for Network Threat Detection in Agriculture 4.0

**DOI:** 10.3390/s21227475

**Published:** 2021-11-10

**Authors:** Nikolaos Peppes, Emmanouil Daskalakis, Theodoros Alexakis, Evgenia Adamopoulou, Konstantinos Demestichas

**Affiliations:** Institute of Communication and Computer Systems, National Technical University of Athens, 15773 Athens, Greece; npeppes@cn.ntua.gr (N.P.); edaskalakis@cn.ntua.gr (E.D.); talexakis@cn.ntua.gr (T.A.); cdemest@cn.ntua.gr (K.D.)

**Keywords:** machine learning, network traffic classification, voting ensemble, network threats, network security, intrusion detection, active attacks, cybersecurity, Agriculture 4.0, e-Commerce

## Abstract

The upcoming agricultural revolution, known as Agriculture 4.0, integrates cutting-edge Information and Communication Technologies in existing operations. Various cyber threats related to the aforementioned integration have attracted increasing interest from security researchers. Network traffic analysis and classification based on Machine Learning (ML) methodologies can play a vital role in tackling such threats. Towards this direction, this research work presents and evaluates different ML classifiers for network traffic classification, i.e., K-Nearest Neighbors (KNN), Support Vector Classification (SVC), Decision Tree (DT), Random Forest (RF) and Stochastic Gradient Descent (SGD), as well as a hard voting and a soft voting ensemble model of these classifiers. In the context of this research work, three variations of the NSL-KDD dataset were utilized, i.e., initial dataset, undersampled dataset and oversampled dataset. The performance of the individual ML algorithms was evaluated in all three dataset variations and was compared to the performance of the voting ensemble methods. In most cases, both the hard and the soft voting models were found to perform better in terms of accuracy compared to the individual models.

## 1. Introduction

Agriculture evolves at a rapid pace nowadays, transitioning into a new era known as Agriculture 4.0. Considering the challenges of modern agriculture (e.g., climate change, diseases, excessive use of chemicals and resources, etc.), Agriculture 4.0 aims to engage new technologies and methods in order to alleviate the existing challenges, reduce the risks and lead to more efficient and safer production. To this end, it engages a plethora of advanced Information and Communication Technologies (ICTs) [1].

In addition to this evolution, there is an upward trend in food needs that, as reported by the United Nations’ Food and Agriculture organization, will reach a 70% increase in 2050 as compared to the current production, so as to cover the needs of the growing population, which is expected to be around 10 billion in 2050 [2,3]. Considering the ongoing evolution and the growing food needs, it is expected that the market size of Agriculture 4.0 will significantly grow in the upcoming years.

In Agriculture 4.0, Wireless Sensor Networks (WSNs) and Internet of Things (IoT) solutions are extensively used, providing numerous benefits to the farmers (e.g., in monitoring various environmental parameters related to the crops, detecting crop diseases, estimating the predicted yield, reducing human labor) [4,5]. However, the interconnection among diverse sensors and network devices, which often contain unpatched or outdated firmware or software [6], in agriculture can create a breeding ground for various attacks (e.g., device attacks, data attacks, privacy attacks, network attacks) [7]. Some examples of network attacks include: malware injection, hacking, phishing, Denial of Service (DoS) attacks, SQL injections and Advanced Persistent Threats (APT) [8,9].

In agriculture, safety is a major concern, and any kind of disturbance or distortion may pose important challenges and lead to dangerous consequences [10,11]. Network traffic monitoring and classification, which have been of great interest since the very early stages of the Internet, can play an important role in the protection from network attacks [12]. Network traffic classification for securing IoT systems has been a subject of intense scientific study. It is a vital component of Intrusion Detection Systems (IDS) and helps to identify and detect malicious network activity [13].

The use of ensemble methods provides an overall better performance in many instances of problems, as compared to the performance of individual classifiers [14]. Based on the above, the key contributions of the current research work can be summarized in three main objectives. The first and foremost is to present and evaluate a hard voting and a soft voting ensemble model comprised of five different ML classifiers (i.e., KNN, SVC, DT, RF and SGD), which are suitable for network attack classifications in the context of Agriculture 4.0 applications. The second objective is to acquire and compare evaluation results of the individual ML classifiers, as well as results on three different variations of the NSL-KDD dataset (i.e., initial dataset, under-sampled dataset, oversampled dataset). The third objective of this paper is to suggest a new line for research works, which evaluates ensemble models of different sets of ML network traffic classifiers on various datasets suitable for Agriculture 4.0 applications.

The remainder of this paper is structured as follows: Section 2 presents an overview of related works in the domain of network traffic monitoring and threats classification for ICTs engaged in Agriculture 4.0; Section 3 demonstrates how a part of the proposed methodology was implemented in the context of an e-Commerce-related EU-funded project; In Section 4 details about the implemented methodology are included; Section 5 focuses on the evaluation results of the models described in Section 4, while Section 6 concludes the paper.

## 2. Related Work

**Figure 1:**
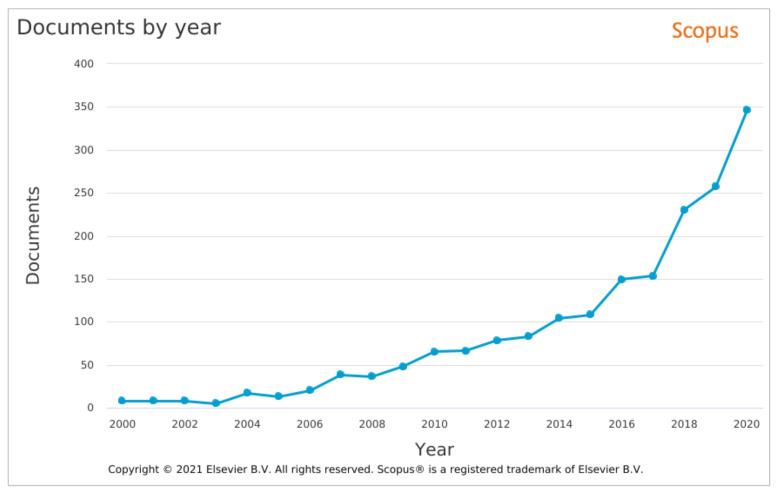
Number of publications in Scopus from 2000 to 2020 [15].

Over the last two decades, both academics and researchers have shown an ever-increasing interest in network traffic monitoring, as depicted in Figure 1. This result was anticipated as the agriculture domain has been becoming more and more digitized, and the ICTs have been evolving rapidly during this period. Over the period 2018–2020, more than 200 new publications appeared in Scopus each year. The same increasing trend is also noticed in 2021, with 286 new publications in Scopus before the end of September. As can be noticed, computer science and engineering come first, followed by agricultural and biological sciences and environmental sciences. The results in Figure 1 were acquired using a query in the Scopus database that searched if any of the below terms is contained in the title, abstract and keywords of publications:

- Network traffic analysis
- Network threats
- Network traffic monitoring
- Network security
- Network intrusion detection
- Network traffic classification

The results were filtered to depict the number of publications between 2000 and 2020 [15].

Aiming at improving security in IoT applications, several ML-based network traffic classification solutions have been proposed. Liu et al. [16] presented a comparative analysis of different ML methodologies in classifying malicious and benign network traffic. Five methodologies were tested, namely Support Vector Machine (SVM), K-Nearest Neighbours (KNN), Logistic Regression (LR), eXtreme Gradient Boosting (XGBoost), and RF. The RF and XGBoost approaches provided the best results in terms of classification performance. Bendiab et al. [17] proposed a solution for network traffic classification that makes use of the visual representation of network traffic data. As a second step, the proposed approach utilized a Residual Neural Network (ResNet50) to analyze the acquired visual data. This methodology was compared to other learning algorithms (i.e., Resnet34, Mobilenet, SOINN) and was found to achieve the best classification performance.

Ullah and Mahmoud [18] presented a two-level flow-based approach for anomaly detection in IoT networks. The first level was responsible for classifying the traffic as normal or anomalous. In case an anomaly was detected, the second level was used to categorize the anomaly. In the context of this model, several ML methodologies were investigated. For the first level, the Decision Tree (DT) classifier was found to achieve the best results in terms of prediction, while the second one, the Random Forest (RF) classifier, was found to yield the best predictive results. In another research work of Ullah and Mahmoud [19], a Convolutional Neural Network (CNN)-based model for anomaly detection in IoT networks was proposed. Based on raw network traffic data, the authors also described a methodology for generating different datasets from an existing one. Transfer learning methods were utilized to generate binary or multi-class network traffic classification models. Experimental results of the proposed model indicated high detection rates in different datasets, combined with a low false alarm rate.

The aforementioned studies mainly refer to IoT network monitoring, as well as to solutions for detecting anomalies, abnormal/malicious behavior and intrusions. Due to the fact that IoT solutions are widely adopted in Agriculture 4.0 [4,5], those solutions can also be relevant and engaged in agricultural IoT networks. A more agriculture-focused study was performed by Ferrag et al. [20], who proposed an Intrusion Detection System (IDS). This IDS is based on three different deep learning models (i.e., Recurrent Neural Networks-RNNs, CNNs and Deep Neural Networks-DNNs), which can be utilized for Distributed Denial of Service (DDoS) attacks in Agriculture 4.0. The authors compared the performance of the aforementioned models to other common ML models (i.e., LR, RFs, Naive Bayes, DTs). Their models were found to perform better than the common ML models, and they even outperformed other state-of-the-art deep learning IDS methodologies [20]. Yin et al. [21] proposed a transformation-based approach, which can be utilized for anomaly detection in agricultural IoT applications. The authors leveraged numerous transformations on raw data samples to achieve improved anomaly detection in a semi-supervised manner. Their experimental results showed that their approach can provide promising results for network flow classification. Towards the same direction, Asonye et al. [22] proposed a network traffic classification methodology aiming to secure ZigBee IoT networks against the HTTP Unbearable Load King (HULK) threat. This methodology can be utilized in smart agriculture applications and encompasses four ML algorithms, i.e., RF, KNN, Naive Bayes and SVMs. The experimental results showed the high effectiveness of the proposed methodology in distinguishing normal traffic from attack traffic [22].

In ML, ensemble classifiers are created by several single estimators (base estimators) that cooperate with each other based on certain training and classification methodologies [23]. Various scientific studies have found ensemble classifiers to provide several advantages over individual classifiers, leading in many cases to more robust classification metrics [24]. In this light, Imran et al. [25] introduced an IDS based on the ensemble of prediction and learning mechanisms to improve abnormal detection accuracy in network environments. Their solution engages a Kalman filter and an Artificial Neural Network (ANN) in order to combine their results in a single output by using a weighted majority method. Gao et al. [26] chose some base classifiers (e.g., DT, RF, KNN, DNN) and designed an ensemble adaptive voting algorithm in order to improve the accuracy results. Their method reached 85.2% accuracy on the NSL-KDD dataset. Rajagopal et al. [27] presented a stacking ensemble solution for network intrusion detection that engages LR, KNN, RF classifiers and an SVM as a metaclassifier after the stacking ensemble process of the aforementioned three with very promising results.

## 3. Implementation of the Proposed Methodology in the Context of the ENSURESEC Project

Several solutions from the e-Commerce domain can also be utilized in the agriculture sector [28]. For example, IoT-based applications for safe transportation of sensitive pharmaceutical products in the context of e-Commerce are relevant to similar solutions for the transportation of sensitive agricultural goods [29,30]. Other popular examples include applications that use ML-based network monitoring tools to detect and classify malicious operations, such as information tampering and DoS attacks. [31,32]. Network traffic classification solutions for IoT systems can also be adapted to be used in agriculture or other sectors since network traffic attributes have similarities across different domains [33].

A part of the current research work has also been implemented in the context of the ENSURESEC project as a subcomponent of a communications monitoring toolset in an e-Commerce environment. The ENSURESEC project has received funding from the European Union’s Horizon 2020 research and innovation program. It aims to protect the whole range of modern e-Commerce by addressing a wide variety of threats. More specifically, it focuses on a wide variety of products ranging from virtual products and services purchased online to physical products bought online and delivered to the customers and aims at addressing numerous threats ranging from e-Commerce web applications attacks to frauds committed by customers or insiders, delivery issues, etc. [34].

The aforementioned toolset offers advanced monitoring capabilities aiming to ensure that the communication protocols in use, as well as the underlying communication infrastructure, function properly and safely. In this direction, certifiably correct verification methodologies (e.g., Decision Tree, Random Forest, KNN, Support Vector Machine, Voting Ensemble) are adopted, and various functionalities are included in the toolset. One of these functionalities is called Threat and Incident Detection and utilizes parts of the methodology described in Section 4. Through this functionality, ENSURESEC users can identify malicious operations or threats at the network level by analyzing, filtering and matching semantically low-level events. Furthermore, they can gain insights into the structured relationships among the various types of items involved.

Advanced threats detection mechanisms are of vital importance for well-designed and secured e-Commerce systems in order to protect and ensure sensitive data that is being targeted by malicious users. Within this scope, the current study evaluates the performance of distinct ML algorithms in Section 5 and provides an additional countermeasure component (Threat and Incident Detection) by engaging two domains of high interest (i.e., ML and cybersecurity). This will help to enhance the analysis of threats patterns and learning from this process in order to detect, prevent and recognize similar types of attacks, enhancing the capabilities of cybersecurity teams to respond in real or near-real time to active types of cyber-attacks.

Five more functionalities were included in the so-called “Communication Monitor” toolset namely:

- A Threat Objects Fusion functionality through which users can fuse different objects into a unified object
- A Similarity Degree Calculation feature that enables users to execute a character-by-character complex comparison algorithm among all the types of objects stored in or retrieved from a specific Knowledge Base, after the execution of certain processes
- An Association Rule Engine that can be used for revealing hidden patterns and relations while exploring a populated database
- A Visualization functionality that can be used for the interactive representation of populated ontologies in the form of graphs
- An Advanced Reasoner through which users can apply rule-based logical reasoning into the existing Knowledge Base

Further analysis of these functionalities is beyond the scope of the current research work.

**Figure 2:**
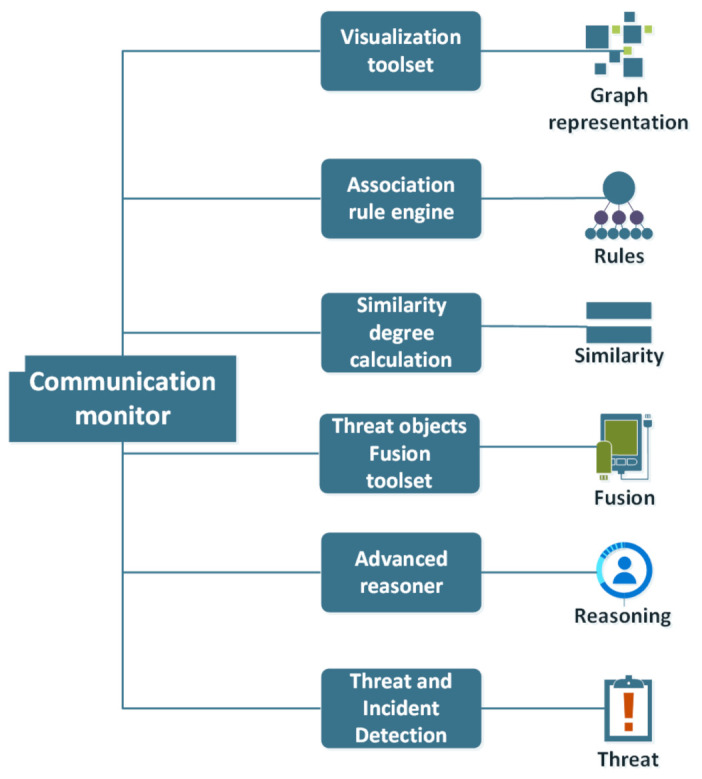
Communication monitor components.

The Threat and Incident Detection functionality, together with the other functionalities of the Communication Monitor, are depicted in Figure 2.

## 4. Dataset and Methodology

Τhis section is divided into two sub-sections; the first sub-section describes the dataset used for the evaluation of the proposed solution, whilst the second focuses on the description of the proposed methodology, i.e., the algorithms and methods engaged to produce the desired results.

### 4.1. Exploring the Used Dataset

For the evaluation needs of this study, the NSL-KDD dataset [35] was used. The NSL-KDD dataset is an evolution of the KDD Cup 1999, which was used for The Third International Knowledge Discovery and Data Mining Tools Competition. The main object of this competition was to create a network intrusion detector model, which could differentiate normal connections from intrusions or attacks [36].

This very task is directly associated with the aim of the methodology analyzed in the context of this paper, which is to detect and classify threats in network traffic. Moreover, the KDD Cup 1999 dataset contains a standard set of data to be audited, which encompasses a wide variety of intrusions. An optimized set of this data, widely used by security professionals, is also contained in the NSL-KDD dataset. There are several distinct types of cyber-attacks; 22 of which are contained in the NSL-KDD training dataset, as presented in Table 1.

**Figure 3:**
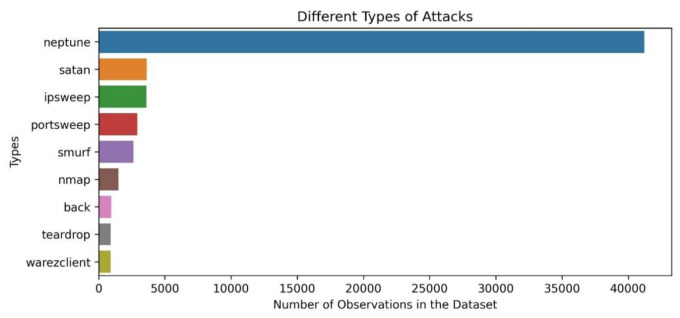
Different types of attacks included in the NSL-KDD dataset [35].

The top ten types of attacks considering the number of records contained in the NSL-KDD training dataset are depicted in Figure 3.

As previously mentioned, the training dataset contains 22 types of attack traffic, whilst the test dataset contains 37 types [35]. The existence of these additional 15 types of attacks was designed with an eye on the generalization of the training dataset in order to test the model’s adjustment capability in unseen types of attacks. The evolution of cyber-attacks was also taken into consideration since cyber-criminals have been seen advancing their capabilities, adjusting quickly and targeting more effectively with the passage of time.

**Figure 4:**
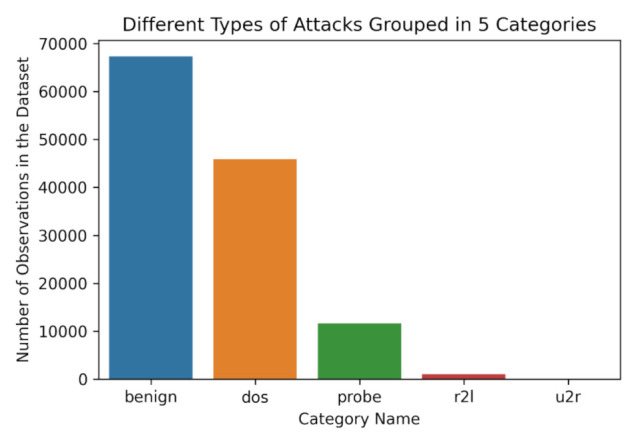
Attack types of NSL-KDD dataset grouped into five different categories [35].

The dataset contains different attack types, as described in Figure 4, that correspond to separate attack categories. The current paper focuses on the utilization of the results from different types of ML Algorithms to categorize each separate instance into one of the below five classes [9,37]:

- ‘benign’ for benign behavior
- ‘dos’ for DoS attempts in an online system
- ‘probe’ for brute-force attack probing by malicious actors
- ‘r2l’ for unauthorized attempts of accessing from remote machines
- ‘u2r’ for Privilege escalation attacks in the target machine

All the instances of the NSL-KDD dataset grouped into five different categories are depicted in Figure 4.

The NSL-KDD dataset optimizations as compared to the original KDD dataset are as follows [35]:

- There are no duplicated/redundant instances, and thus, the classifiers will not be biased by these records
- Every difficulty level group contains a number of instances that is inversely proportional to the percentage of instances in the KDD Cup 1999 dataset. This leads to a wide range of classification rates of the distinct ML methods. Thus, the evaluation of different ML algorithms can be more accurate
- The size of both the train and the test set is configured in order to offer the ability to whoever is interested in running their experiments on commercially available PCs. Therefore, the direct comparison of different research efforts that used the NSL-KDD dataset is feasible

One important issue detected in the NSL-KDD dataset that can affect the performance of the suggested classifiers is the unbalanced data, commonly known as a class-imbalance problem. As Chawla et al. [38] stated, the class imbalance problem constitutes a new major problem for the data mining community, whereas a large part of the research community is continuously investigating ways to reduce this. Dealing with the class imbalance problem is a cutting-edge research topic [39]. Chawla et al. [40], as well as Bowyer et al. [41], proposed a number of solutions at both the algorithmic and the data levels, including different oversampling and undersampling methods. In general, undersampling as a term is relevant to the reduction of the number of the samples, whilst the oversampling term refers to the generation of synthetic data instances based on the classes with the least instances. Many different strategies of data resampling have been considered by the research community, including [42]:

- Random undersampling of the majority class of the used dataset
- Directed undersampling of the informed class of the used dataset
- Oversampling with replacement of the minority class of the used dataset to generate a more balanced dataset
- Directed oversampling, where specific dataset information is created and replaced in a non-random manner
- Adjusting the costs of the various dataset’s classes
- Adjusting the decision threshold
- Adjusting the estimation of the probabilistic factor at the tree leaf, mostly in cases where decision trees algorithms are involved

To avoid negative side effects stemming from the use of distinct methods, different types of resampling methods can be combined. For the purposes of the current study, the “random undersampling” and the “oversampling with replacement of the minority class” data resampling techniques are adopted. These techniques are applied individually in each distinct algorithm in order to highlight the differences between the datasets that have undergone resampling and the initial, raw imbalanced dataset. Table 2 depicts the original (training) dataset distribution alongside the oversampled and the undersampled results.

### 4.2. Proposed Methodology

In recent years, ensemble learning models are becoming increasingly popular among researchers [43] in the field of predictive modeling, i.e., regression and classification analysis. The term voting ensemble is used to describe an ensemble ML model based on the combination of multivariate ML models, aiming to achieve a better performance compared to the performance of each individual model, leading to an overall improved classification performance [44,45]. More specifically, the combination of multiple ML models with similar performance on a predictive task can eventually lead to models with higher accuracy and fewer errors.

Two popular voting ensemble methods are (i) hard voting, which is suitable for models that predict distinct class labels in cases where the outputs of classifiers are not independent, as well as for binary class problems when the number of included classifiers is not odd, and (ii) soft voting, which is mostly used in cases where the developed models predict probabilities for each contained class, as well as when it is clear that a classifier returns better output results in comparison to the other classifiers included [46]. More specifically, the hard voting method takes the predictions from each individual classifier as input and then calculates the votes for each target label. After this calculation, the label with the majority of the votes is the prediction/result of the hard voting ensemble model. Assuming we have a binary problem and five classifiers, then considering that four of them predict/vote for the class labeled “1” and the other classifier votes for a class labeled “0” then the ensemble model decision is the class labeled “1” supported by the 4/5 of the votes. As far as the soft voting is concerned, it is a more complex method that takes into account the probability of predictions by the individual classifiers. For example, assuming again we have five classifiers and the prediction for a class labeled “1” has probability 80% by classifier 1, 72% by classifier 2, 67% by classifier 3, 63% by classifier 4 and 49% by classifier 5, then the average of these probabilities is 66.2% to be class labeled “1”, thus the soft voting method result is class labeled “1”. Other popular ensemble strategies that can be found in the scientific literature are described below [47]:

- Boosting (e.g., AdaBoost)
- Bagging
- Stacking or stacked generalization
- Blending

**Figure 5:**
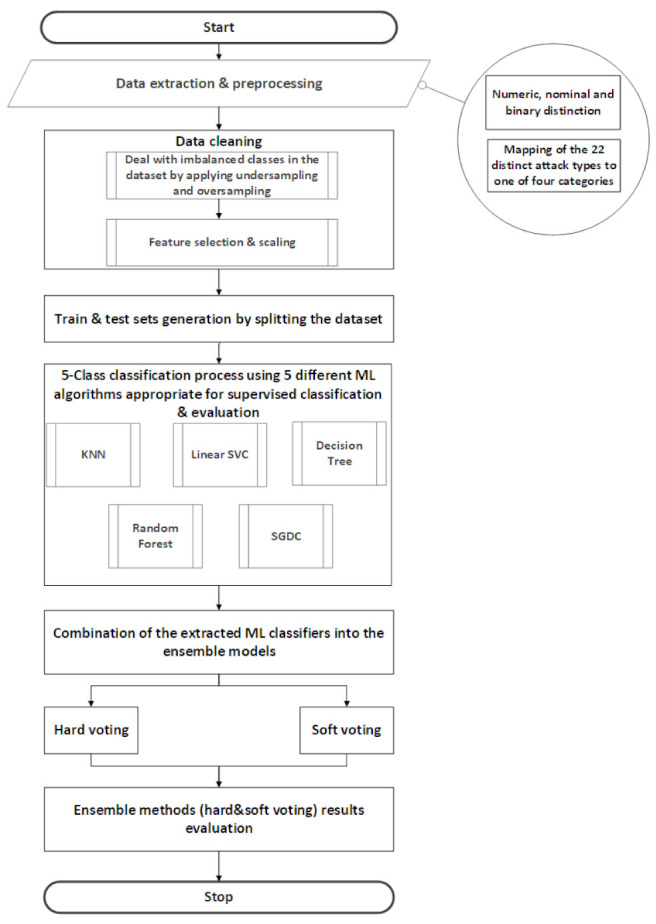
Methodology steps taken for this study.

The methodology proposed in this study aims to evaluate hard voting and soft voting ensemble methods. These two methods encompass five different ML algorithms namely; KNN, linear SVC, DT, RF and SGD, as shown in Table 3. Each of the five classifiers was evaluated individually in order to highlight the performance differentiations among the classifiers, as well as the improvements of the ensemble model in terms of accuracy. A more detailed description of the steps performed can be found in Figure 5.

KNN is one of the most studied supervised ML classifiers. KNN uses distance metrics (i.e., Euclidean, Manhattan and Minkowski functions) in order to classify each data instance to its nearest neighborhood [48,49]. For the purposes of this study, the K parameter is equal to 7. Linear SVC is a variation of the SVM algorithms. SVM is a supervised ML algorithm that aims to find a hyperplane in an N-dimensional space, where N is the number of features, so as to classify the data points accordingly. For network threat detection, a linear SVM can classify the network traffic into benign and threat or abnormal classes [50]. In this study there is an evaluation of the linear SVC in terms of accuracy. The DT algorithm is a non-parametric supervised learning method used for classification purposes. It is characterized by “if-then-else” rules, and its goal is to classify a data instance into the most suitable class, depending on its attributes. Thus, when a dataset is given as an input to a DT, the algorithm learns the patterns and the attributes of the given data and can detect threats and abnormal behavior [51,52]. The RF method can be considered as an ensemble approach of several DT instances. The RF algorithm inherits some features of the DT method and is mainly used for classification problems. As is indicated by its name, RF utilizes the power of the crowd of the separate DTs, and its output is the class or the category selected by a majority of the trees [53]. Based on the above, RF can be used to classify network operations in different categories so as to detect threats and possibly malicious behavior [54]. Finally, the SGD classifier was engaged for the needs of this study. The SGD algorithm can be considered as an optimization method for linear classifiers, such as the SVM. More specifically, the SGDC method implements a stochastic gradient descent routine that is enabled to support different loss functions and penalties. Thus, an SGDC implementation draws a boundary plane that classifies the data instances [55].

## 5. Results

The algorithms were developed in Python version 3.8, and the sci-kit learn framework [56,57] was utilized for the development of the proposed algorithms together with the imblearn framework [58], which was used for resampling processes on the imbalanced dataset. The parameters and their values for each individual classifier, as well as for the two ensemble models, are depicted in Table 4.

Each of the algorithms introduced in Section 4, as well as the ensemble methods (hard and soft voting), are evaluated using the accuracy metric. The accuracy metric is practically the number of correct predictions divided by the total number of predictions made, as shown in Equation (1) [59]. In this study, the accuracy is calculated as the number of the correctly classified data instances by each algorithm divided by the number of the total samples contained in the dataset, as described in Section 4.1.

(1) $$accuracy=\frac{TP+TN}{TP+TN+FP+FN}$$

where

- *TP* stands for True Positives, i.e., the attack predictions that are actually attacks
- *TN* stands for True Negatives, i.e., the non-attack predictions that are actually non-attacks
- *FP* stands for False Positives, i.e., the attack predictions that are not actually attacks
- *FN* stands for False Negatives, i.e., the non-attack predictions that are actually attacks

The calculated accuracy results for the three different variations of the dataset (initial dataset, undersampled dataset and oversampled dataset) for every method used (i.e., five classifiers and two ensemble methods) are presented in Table 5.

**Figure 6:**
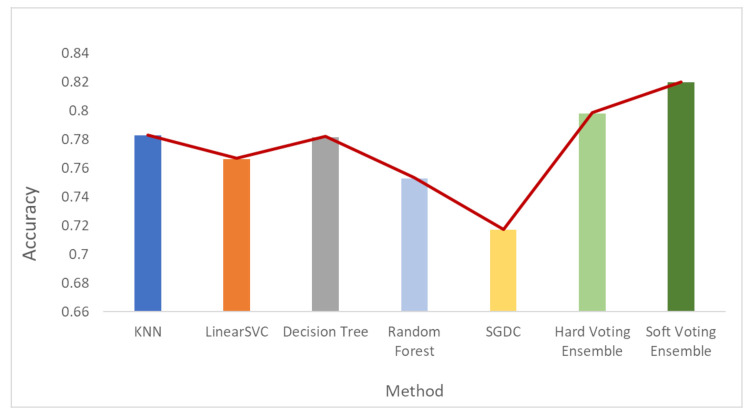
Accuracy results when the initial dataset was used.

**Figure 7:**
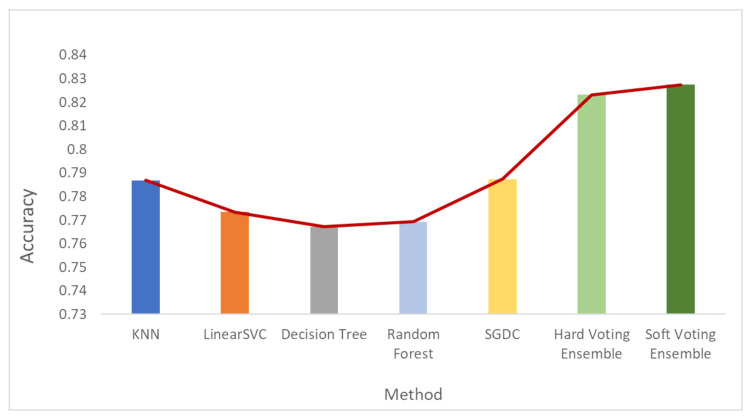
Accuracy results when the undersampled dataset was used.

**Figure 8:**
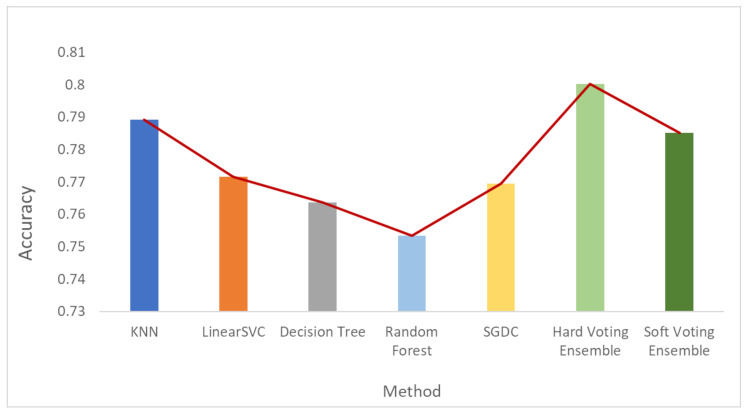
Accuracy results when the oversampled dataset was used.

Figure 6, Figure 7 and Figure 8 depict the accuracy results regarding each variation of the used dataset (initial dataset, undersampled dataset and oversampled dataset).

**Figure 9:**
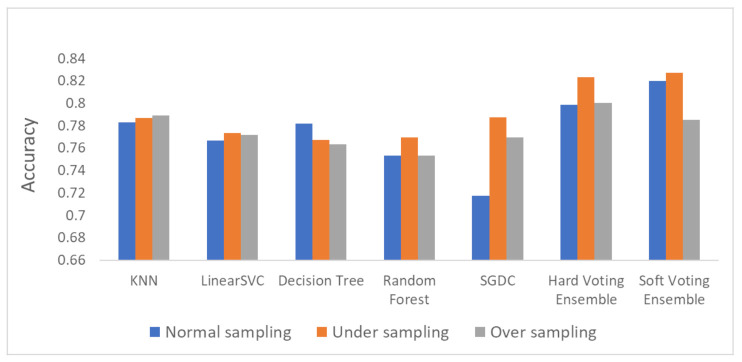
Overall accuracy results.

**Figure 10:**
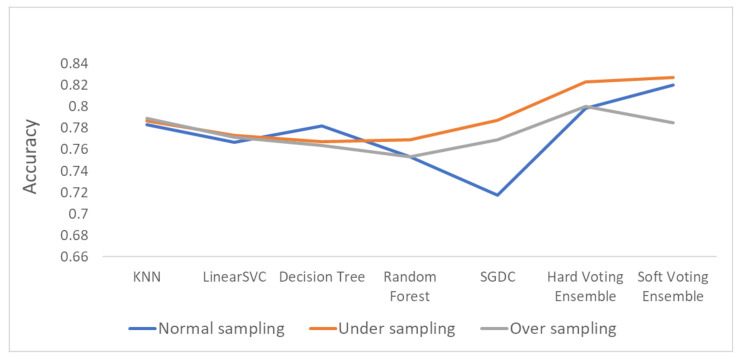
Linear plot for the accuracy results.

The overall accuracy results are presented in Figure 9 (bar plot) and Figure 10 (linear plot) below.

Figure 10 includes a linear plot for the accuracy of all the methods and datasets that were evaluated.

Based on the results presented in the above figures, as well as in Table 5, useful conclusions can be drawn. Τhe accuracy metrics of the performed ensemble algorithms are quite promising compared to the accuracy of each individual classifier. More specifically, the hard voting model was found to achieve better accuracy in all three dataset variations used (initial dataset, oversampled dataset and undersampled dataset) compared to the accuracy of the individual classifiers on the same datasets. The soft voting model achieved better accuracy than the individual classifiers in both the initial and the undersampled datasets. It also yielded better accuracy results than the hard voting model in these two dataset variations. Using the initial dataset, which had not undergone any resampling process, the highest accuracy was achieved by the soft voting ensemble model, and the lowest was achieved by the SGDC algorithm. Using the undersampled dataset, the highest accuracy was achieved by the soft voting ensemble model, and the lowest was achieved by the DT algorithm. Finally, when the oversampled dataset was used, the highest accuracy was achieved by the hard voting ensemble model, whilst the lowest was achieved by the RF algorithm implementation.

It is worth mentioning that the model with the highest accuracy, among all the algorithms and datasets (normal or resampled) combinations, was the soft voting ensemble model using an undersampled dataset, while the SGDC algorithm, using the initial dataset had the lowest accuracy.

Another interesting remark based on the aforementioned results is the increased accuracy that was noticed by adopting the selected dataset resampling methods. Except for the DT algorithm, where the normal sampling was found to achieve higher accuracy compared to the two resampling methods, in the rest of the six algorithm implementations, datasets that had undergone resampling achieved better classification performance.

## 6. Conclusions

The goal of this study was to present a comparative analysis of the performance of five different ML classifiers when they are applied individually, as well as when they are part of hard and soft voting ensemble methods. For the evaluation of these methods, the NSL-KDD dataset was utilized in three variations (i.e., initial/normal dataset, undersampled dataset and oversampled dataset). Hard voting and soft voting ensemble models were found to achieve a better overall accuracy than the individual classifiers in most cases.

The analyzed solutions can be used for network traffic classification in the context of Agriculture 4.0. To this end, this paper includes a related works section where several studies about network traffic monitoring and classification were presented. These studies refer to contemporary solutions that are directly connected with the agriculture domain or the ICT technologies that are engaged in it. In the agriculture sector, cyber-security is of utmost importance, so the engagement of new tools and technologies, including ensembles of ML methods, can contribute to a safer and more robust network infrastructure encompassing a plethora of devices.

Details were also provided about how the analyzed methodology was implemented in the context of a communications monitoring toolset in an e-Commerce related EU-funded project.

The use of ensemble ML models is an expanding research topic, and new possibilities are explored in order to enhance the capabilities and the performance of single ML classifiers in this domain. The results presented in this study seem to be very promising concerning the utilization of ensemble methods in network traffic classification. A future step of this analysis would be to integrate it in an application where historical and near real-time analyses for network attacks classification are required so as to detect threats and abnormal traffic in order to isolate such traffic and/or provide alerts. Furthermore, we propose that the evaluation of the performance and efficiency of such models is also performed in other datasets and domains. We also suggest the examination of different ensemble models other than hard and soft voting. Another useful approach would be to engage different ML classifiers and combine various solutions in order to investigate the best option. Furthermore, the engagement of such methods may be expanded in different domains other than agriculture and e-Commerce in order to discover new possibilities and restrictions imposed by different kinds of datasets.

## Figures and Tables

**Table 1:** Types of attacks in the NSL-KDD dataset [35].

Types of Attacks	
neptune	satan
ipsweep	portsweep
smurf	nmap
back	teardrop
warezclient	pod
guess_passwd	buffer_overflow
warezmaster	land
imap	rootkit
loadmodule	ftp_write
multihop	phf
perl	spy

**Table 2:** Records of each category included in the three variations of the dataset [35].

Dataset Name/Classifier	Benign	dos	Probe	r2l	U2r
Original Dataset	67,343	45,927	11,656	995	52
Undersampled Dataset	25,194	25,194	25,194	25,194	25,194
Oversampled Dataset	67,343	67,343	67,343	67,343	67,343

**Table 3:** Hard and soft voting base classifiers ensemble.

Methodology	Classifier 1	Classifier 2	Classifier 3	Classifier 4	Classifier 5
Hard voting	KNN	Linear SVC	Decision Tree	Random Forest	SGDC
Soft voting	KNN	Linear SVC	Decision Tree	Random Forest	SGDC

**Table 4:** Individual ML classifiers and ensemble models parameters.

	Classifier	Parameters
Machine Learning Algorithms	KNN	n_neighbors: 7, n_jobs:5, weights:“uniform”, algorithm:“auto”
	Linear SVC	C: 1.0, kernel: ‘linear’, degree:1, gamma: ‘scale’, class_weight: ‘balanced’, decision_function_shape: ‘ovr’, random_state:42
	DT	criterion: “gini”, splitter:“best”, random_state:42
	RF	n_estimators: 200, criterion: ‘entropy’, max_features: ‘auto’, class_weight: ‘balanced’, random_state:42
	SGDC	loss:“hinge”, penalty:“l2”, alpha:“1e-3”, max_iter:5, tol:“None”, random_state:42
Ensemble Algorithms	Hard voting	voting:“hard”, weights: “None”, n_jobs:5, flatten_transform:“True”
	Soft voting	voting:“soft”, weights: “None”, n_jobs:5, flatten_transform:“True”

**Table 5:** Accuracy results for every algorithm and dataset tested.

Dataset	ML Method						
	KNN	Linear SVC	Decision Tree	Random Forest	SGDC	Hard Voting Ensemble	Soft Voting Ensemble
Normal sampling	0.78317423	0.76685060	0.78210965	0.75341554	0.71741661	0.79875798	0.82014580
Undersampling	0.78692317	0.77346851	0.76721097	0.76934416	0.78741661	0.82318758	0.82739580
Oversampling	0.78927434	0.77164506	0.76360965	0.75341554	0.76941661	0.80019798	0.78517984

## Data Availability

The data presented in this study is contained in the NSL-KDD dataset that is publicly available. The resampled datasets described in the study are also available on request from the corresponding author.
